# Mechanism of Carbon Diffusion and Phase‐Transition‐Induced FCC‐TiC Formation via Hot Pressing

**DOI:** 10.1002/advs.202511724

**Published:** 2025-10-07

**Authors:** Sheng Zeng, Guoqiang You, Yuefeng Ying, Lin Deng, Cheng Gu, Bin Jiang

**Affiliations:** ^1^ College of Materials Science and Engineering Chongqing University Chongqing 400045 China; ^2^ National Engineering Research Center for Magnesium Alloys Chongqing University Chongqing 400044 China; ^3^ National Key Laboratory of high‐end equipment casting technology Chongqing University Chongqing 400044 China

**Keywords:** FCC‐TiC, formation mechanism, octahedral interstice, steel–Ti composite structure

## Abstract

Titanium carbide (TiC), which typically forms at the solid‐state diffusion interface in steel–titanium (Ti) composite structures, significantly influences steel–Ti interface bonding. However, the atomic‐level formation mechanism of this carbide remains unclear. Herein, the TiC crystal structure and formation mechanism are investigated through experiments involving TA2 pure Ti and 45# carbon steel under solid‐state diffusion conditions. Analysis of the solid‐state diffusion behavior between the steel and Ti, based on selected area electron diffraction, reveals the formation of a continuous micro–nano TiC layer with a balanced (FCC) crystal structure on the substrate near the Ti side of the interface. Using the integrated differential phase contrast technique, occupation of the octahedral interstices in the FCC‐TiC lattice by carbon (C) atoms is confirmed for the first time. Additionally, it is suggested that C diffusion and phase transformation jointly induce the FCC‐TiC crystal phase transformation under hot‐pressing conditions. Finally, the atomic‐scale TiC formation mechanism is elucidated. The findings of this study may guide the design and development of high‐performance materials with unique properties for aerospace equipment manufacturing.

## Graphical abstract

A solid‐state diffusion bonding steel‐titanium interface is designed, revealing the formation of continuous micro–nano‐sized FCC‐TiC grains at the interface. Carbon atoms occupying the octahedral interstice in the FCC‐TiC lattice are directly observed. The study shows that FCC‐TiC formation is driven by carbon diffusion and titanium phase transformation, providing new insights into atomic‐scale regulation of titanium carbide.

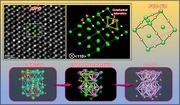

## 1 Introduction

Steel–titanium (Ti) composite structures exemplify a prominent form of dissimilar metal joining, which is typically accomplished using solid‐state bonding techniques such as explosive, roll, and diffusion bonding.^[^ ^1^, ^2^, ^3^, ^4^, ^5^, ^6^, ^7^, ^8^, ^9^, ^10^ ^]^ These composite structures effectively integrate the high strength and cost‐effectiveness of steel with the corrosion resistance and high specific strength of Ti. Consequently, they are extensively utilized in the aerospace, military, petrochemical, and bridge construction sectors.^[^ ^11^, ^12^, ^13^, ^14^ ^]^ During the fabrication process, elevated temperatures and pressures enhance the interfacial contact between steel and Ti, thereby facilitating metallurgical bonding through mutual diffusion. However, multiple studies^[^ ^5^, ^15^, ^16^, ^17^, ^18^, ^19^ ^]^ have indicated that the interfacial reactions in such systems comprise both iron (Fe)/Ti interactions as well as chemical reactions between the carbon (C) within steel and Ti, causing the formation of titanium carbide (TiC). Further, when the temperature or C content of the steel is increased, the chemical reaction between the C and Ti intensifies^[^ ^16^, ^18^, ^20^ ^]^; this behavior likely occurs because the C atoms exhibit higher diffusion rates under high‐temperature conditions owing to their small atomic radius. Notably, during the solid‐state fabrication process, the C atoms compete with Fe and preferentially react with Ti, thereby inhibiting the diffusion and reaction of Fe. Moreover, when the C content of steel exceeds 0.01%,^[^ ^16^, ^18^ ^]^ a TiC phase emerges at the interface. This pronounced interaction between Ti and C has attracted considerable attention.

TiC is a stable transition‐metal carbide in which C and Ti atoms interact through both ionic and covalent bonds, providing a system with strong chemical bonding.^[^ ^21^, ^22^ ^]^ This characteristic imparts excellent physical and chemical properties to TiC, including extremely high hardness, wear resistance, and significant electrical and thermal conductivities. Furthermore, TiC exhibits good chemical and thermal stability with oxidation resistance in high‐temperature, high‐pressure, and corrosive environments. These attributes render TiC a key material for aerospace, industrial manufacturing, and new energy applications.^[^ ^23^, ^24^ ^]^ As the C‐to‐Ti atomic‐radius ratio is relatively small, TiC is classified as an interstitial phase.^[^ ^25^, ^26^ ^]^ TiC exhibit a face‐centered cubic (FCC) crystal structure; however, it is unclear how Ti, which initially has a hexagonal close‐packed (HCP) or body‐centered cubic structure (below and above the phase‐transition temperature, respectively), transforms into the FCC structure of TiC. Most researchers believe that this structural transformation can be attributed to the high thermodynamic stability of FCC‐structured TiC. Consequently, associated studies have predominantly focused on the morphological distribution of TiC.^[^ ^5^, ^15^, ^16^, ^17^, ^20^, ^27^, ^28^, ^29^ ^]^ However, the TiC formation mechanism under solid‐state conditions remains unclear.

This study investigates the inevitable formation of TiC during the solid‐state fabrication of a steel–Ti diffusion couple comprising TA2–45# steel. A solid‐state diffusion bonding method is used to regulate the TiC formation through interfacial diffusion reactions under high‐temperature and pressure conditions. Focusing on the TiC formation mechanism at the interface of the diffusion couple, this study aims to provide key scientific evidence elucidating the reaction mechanism at the steel–Ti interface that enables strength and toughness tuning.

## 2 Results and Discussion

**Figure 1:**
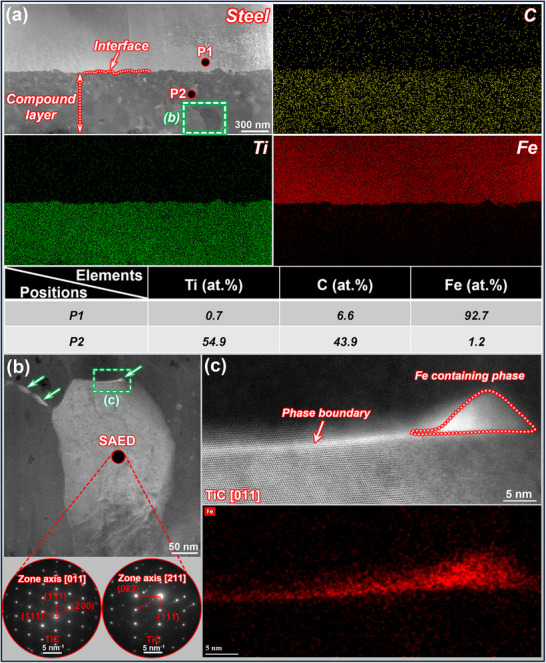
Morphology and EDS mapping results of steel–Ti interface: a) morphological characteristics of the interface, b) TiC grains within the compound layer, and c) Fe distributed along the TiC grain boundary.

The morphology and energy spectrum of the steel/Ti interface following holding at 840 °C and 35 MPa for 80 min are obtained through the high‐angle annular dark field (HAADF) mode (**Figure** **1**). The interface position was determined from the differences in contrast on either side of the microstructure (Figure 1a) and the corresponding scanning results. The interface exhibits nonlinear and continuous characteristics. Elemental distribution analysis for C, Ti, and Fe reveals significant diffusion of C from the steel side to the Ti side, which corresponds to the Ti energy spectrum, along with comparatively minimal mutual diffusion between the Ti and Fe. Point‐scanning analyses conducted at different positions (P1 and P2) near the interface indicated that P1 primarily contains Fe, which corresponds to the original steel base material. However, P2 below the interface is primarily comprised of Ti and C. These results suggest that C and Ti react chemically, causing the formation of a continuous Ti–C compound layer on the original Ti substrate. The phase characterization of this compound layer is shown in Figure 1b. Selected area electron diffraction (SAED) patterns of the various crystal axes confirm that this phase comprises TiC, as shown in Figure 1 (b, lower). Interestingly, no discernible Ti–Fe phase is detected within the compound layer; however, small quantities of white strip‐like substances are observed at the TiC grain boundaries (indicated by the green arrows in Figure 1b). According to the mapping scan results shown in Figure 1c, these white strip‐like substances are Fe‐rich, indicating that Fe diffusion is somewhat inhibited by the continuous TiC layer,^[^ ^16^, ^19^ ^]^ resulting in the diffusion of a small amount of Fe along the TiC grain boundaries.

**Figure 2:**
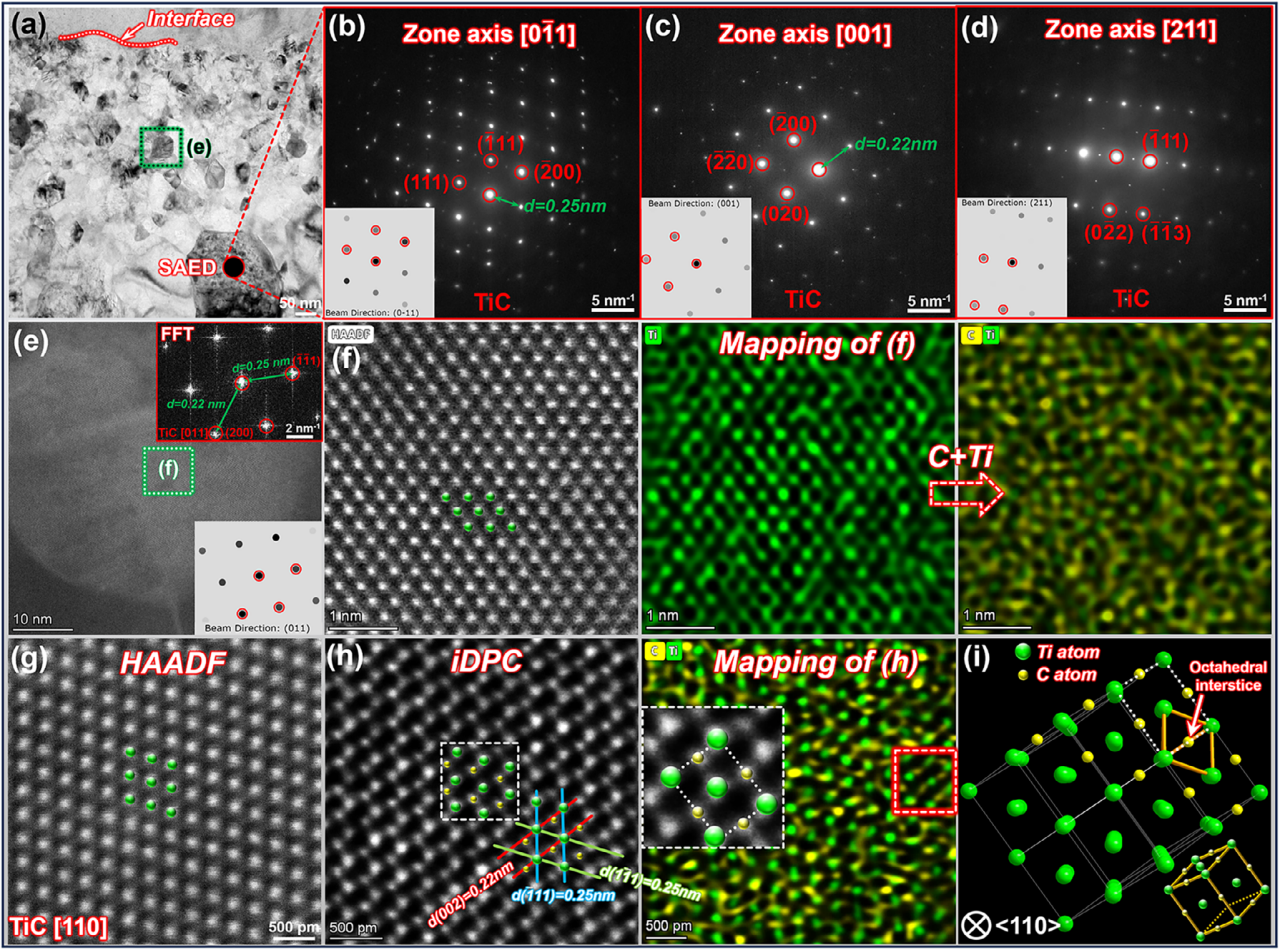
Morphology of steel–Ti interface compound layer and TiC crystal structure under AC‐TEM: a) morphological characteristics of compound layer; b–d) SAED patterns of the same TiC phase along three different zones; e) HAADF image of TiC; f) HAADF atomic image within TiC crystals; g,h) comparisons of HAADF and iDPC atomic images within TiC crystals; i) theoretical model of TiC atomic structure.

As TiC is the main component of the interface, analyzing its microstructural characteristics is critical to elucidate the interface formation mechanism, its crystal‐structure features, and atomic occupancy, particularly that of C atoms. **Figure** **2**a shows the formation of nanometer‐to‐submicrometer‐size TiC crystals within the compound layer. SAED patterns of the same TiC phase below the interface were obtained along three different zone axes, as shown in Figure 2b–d. These patterns were then compared with the FCC‐TiC structure provided in the Materials Project (MP) database (mp‐631).^[^ ^30^ ^]^ (The standard diffraction patterns corresponding to the crystal zone axis are shown in the lower left corner of Figure 2b–d). Based on the analysis using CrysTBox^[^ ^31^ ^]^ crystallographic software, the axes of the three diffraction patterns are determined to be [0$\bar{1}$1], [001], and [211], and the interplanar spacings *d* of the (111) and (020) crystal planes are measured as 0.25 and 0.22 nm, respectively. To quantitatively analyze the relative error, the lattice constant of the TiC phase obtained from the experiment was compared with the standard FCC‐TiC lattice constant (*a* = 0.433 nm) provided in the MP database. The *a* value of the cubic crystal system can be calculated using the following equation:^[^ ^32^ ^]^

(1) $$a=d_{\mathrm{hkl}}\;\cdot \;\sqrt{h^{2}+k^{2}+l^{2}}$$

here *a* is the lattice constant; *d_hkl_* is the spacing between adjacent (*hkl*) planes; and *h*, *k*, and *l* are the Miller indices. For the (111) plane, *d*_111_ is measured as 0.25 nm, with *h* = *k* = *l* = 1. Similarly, for the (020) plane, *d*_020_ is measured as 0.22 nm, where *h* = *l* = 0 and *k* = 2. Using Equation (1), the TiC *a* values can be calculated as 0.433 and 0.440 nm, respectively, corresponding to relative errors of 0 % and 1.6 % compared with the standard value. This indicates that the observed phase is in thermodynamic equilibrium and has an FCC‐TiC crystal structure. To investigate the TiC atomic occupancy in detail, aberration‐corrected transmission electron microscopy (AC‐TEM) was applied to another TiC phase, as shown in Figure 2e. A fast Fourier transform image is shown in the upper‐right corner of Figure 2e; the observation direction corresponds to that of the <110> crystal family of the FCC structure. The spacings of the {111} and {200} crystal plane families are measured as 0.25 and 0.22 nm, respectively. These results indicate that TiC phases at different positions within the compound layer exhibit the same thermodynamic state and crystal structure. Moreover, the Ti atom arrangement along the [110] crystal axis in the TiC lattice is confirmed by HAADF images and atomic‐level mapping scan results, as shown in Figure 2f. Each pair of adjacent Ti atoms in the horizontal direction forms a rhombic structure with the nearest two Ti atoms in the subsequent row. However, because of the light nature of C atoms and their significantly weaker electron scattering ability than that of Ti atoms, the HAADF mode provides limited‐quality imaging for C atom occupancy information. Consequently, the integrated differential phase‐contrast (iDPC) technique was used to image the same positions within the TiC lattice. As apparent from the figures, only Ti atoms are visible in the HAADF mode (Figure 2g), whereas both Ti and C atoms are imaged simultaneously in the iDPC mode (Figure 2h). In combination with the mapping scan results of the red‐box area, the iDPC method clarifies the C atom arrangement in the TiC lattice. Using this strategy, the C and Ti atom occupancy in the TiC lattice is experimentally observed for the first time. Compared with the theoretical model of TiC shown in Figure 2i, the C atoms in the equilibrium‐state TiC lattice are positioned in the octahedral interstices of the FCC lattice, whereas the Ti atoms occupy the unit cell and face‐centered positions of the FCC structure. These results provide a theoretical basis for elucidating the TiC formation mechanism.

Based on the experimental results, we determined the reaction products and characteristics of the crystal structure at the steel–Ti interface following hot pressing. The compound layer at the interface was characterized as a continuous micro–nano‐sized FCC‐TiC phase formed by a chemical reaction between C and Ti. Moreover, the SAED patterns of different zone axes are consistent with those given for TiC (mp‐631) in the MP database. The use of iDPC facilitates the direct observation of the Ti and C atom arrangements within the FCC‐TiC lattice. These results were used to propose a process through which HCP‐Ti transitions to FCC‐TiC, as this process has not been explored previously. It is hypothesized that the transformation is thermodynamically driven by the free energy difference between the phases, while the energy supplied during hot pressing primarily serves to overcome the activation energy barrier. Therefore, two hypotheses regarding the FCC‐TiC formation mechanism are proposed and discussed in this study: first, hot pressing induces the Ti phase transformation from the HCP to FCC lattice, after which C atoms occupy the octahedral interstitial sites to form FCC‐TiC; second, the interatomic interactions caused by C diffusion may drive the crystal structure transformation from HCP to FCC.

**Figure 3:**
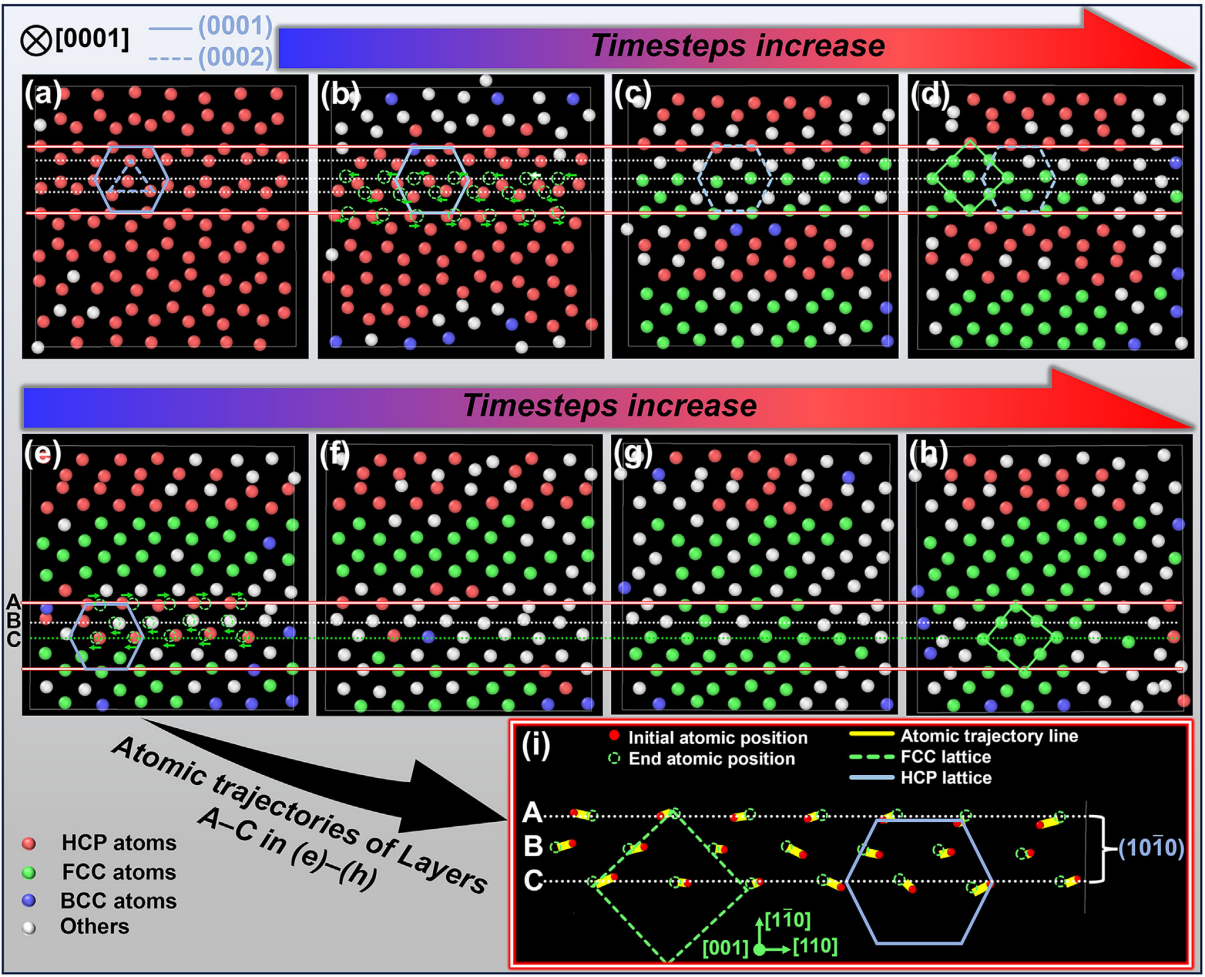
a–h) Molecular dynamics simulation of HCP to FCC structural transformation induced by thermal pressure, i) trajectory lines of A/B/C three‐layer atoms during transformation.

First, an HCP‐Ti crystal model was constructed using molecular dynamics simulations (via LAMMPS) and used to investigate the combined effect of temperature and pressure on the Ti phase transformation under the experimental conditions. The crystal structure evolution was visualized using OVITO software. The evolution of the Ti crystal structure along the [0001] direction (i.e., along the direction of the applied loading force) revealed an ABAB pattern for the atomic stacking sequence characteristics of the HCP crystal structure (**Figure** **3**). To differentiate between atoms across various crystal planes, the following specific marking convention was used: solid lines within a hexagon were used to connect atoms of the A layer (0001) crystal plane, whereas dashed lines were used to connect atoms within the B layer (0002) crystal plane (Figure 3a). Figure 3a–h shows the HCP‐to‐FCC phase‐transformation process across different regions. Taking Figure 3a–d as a case study, the atomic displacement behavior was observed by marking specific areas with horizontal lines. With increasing time steps, the atoms in this region transition from the HCP to the FCC structure.

We next outline the key transformation process. In Figure 3b, the dashed circles and green arrows indicate the positions and displacement directions, respectively, of the atoms in the subsequent step. Atoms from the (0001) crystal plane in the HCP structure gradually occupy positions within the FCC unit cell, whereas those from the (0002) crystal plane shift toward the face‐centered positions in the FCC crystal. Notably, the atoms situated between the crystal planes in the original HCP structure undergo displacement in opposite directions. Similar displacement patterns are apparent in Figure 3e–h. The displacement trajectories of the atoms in Layers A, B, and C, shown in Figure 3e, were exported through OVITO, as shown in Figure 3i. With the structure shown in Figure 3e being taken as the initial position, the yellow solid line in Figure 3i indicates the atomic motion trajectory. Figure 3i indicates that the atoms in Layer B in the HCP structure tend to migrate toward the face‐centered positions in the FCC structure. Conversely, the atoms in Layers A and C on the (10$\bar{1}$0) crystal plane was displaced in the opposite direction along the [11$\bar{2}$0] axis, ultimately forming the (110) crystal plane in the FCC structure. Throughout this process, the FCC lattice constant *a* increases and the lattice spacing expands, facilitating the accommodation of C atoms.

**Figure 4:**
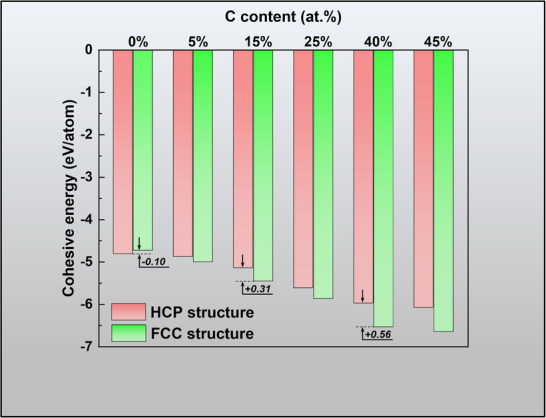
Cohesive energies of HCP and FCC structures under different C doping conditions.

The second assumption is that the diffusion of C atoms facilitates the formation of the FCC phase. According to Jia et al.,^[^ ^33^ ^]^ C atoms can inhibit the FCC‐to‐tetragonal structural transformation. Therefore, the expectation that C atoms induce an HCP‐to‐FCC structural transformation is reasonable. To investigate this hypothesis, HCP and FCC supercells were separately constructed using Atomsk modeling software, and varying concentrations of C were doped into the octahedral interstices of the structures. A comparison of the cohesive energies of the FCC and HCP crystal structures under different C‐doping conditions is shown in **Figure** **4**. In the absence of C doping, the stability of the HCP structure exceeds that of the FCC structure. However, with increasing C content, the cohesive energies of both the HCP and FCC structures gradually decrease, suggesting that the introduction of C atoms enhances the stability of both structures. Notably, the cohesive energy of the FCC structure is more negative than that of the HCP structure, indicating greater stability. Furthermore, the difference in cohesive energy between the two structures increases with increasing C content, indicating that the introduction of C atoms renders the FCC structure more stable than the HCP structure. This phenomenon can be attributed to the interaction between the Ti and C atoms. Because of the difference in symmetry between the HCP and FCC lattices, the atomic interactions vary accordingly. As the C content increases, it facilitates the formation of the FCC structure, thereby reducing the total energy of the system.

**Figure 5:**
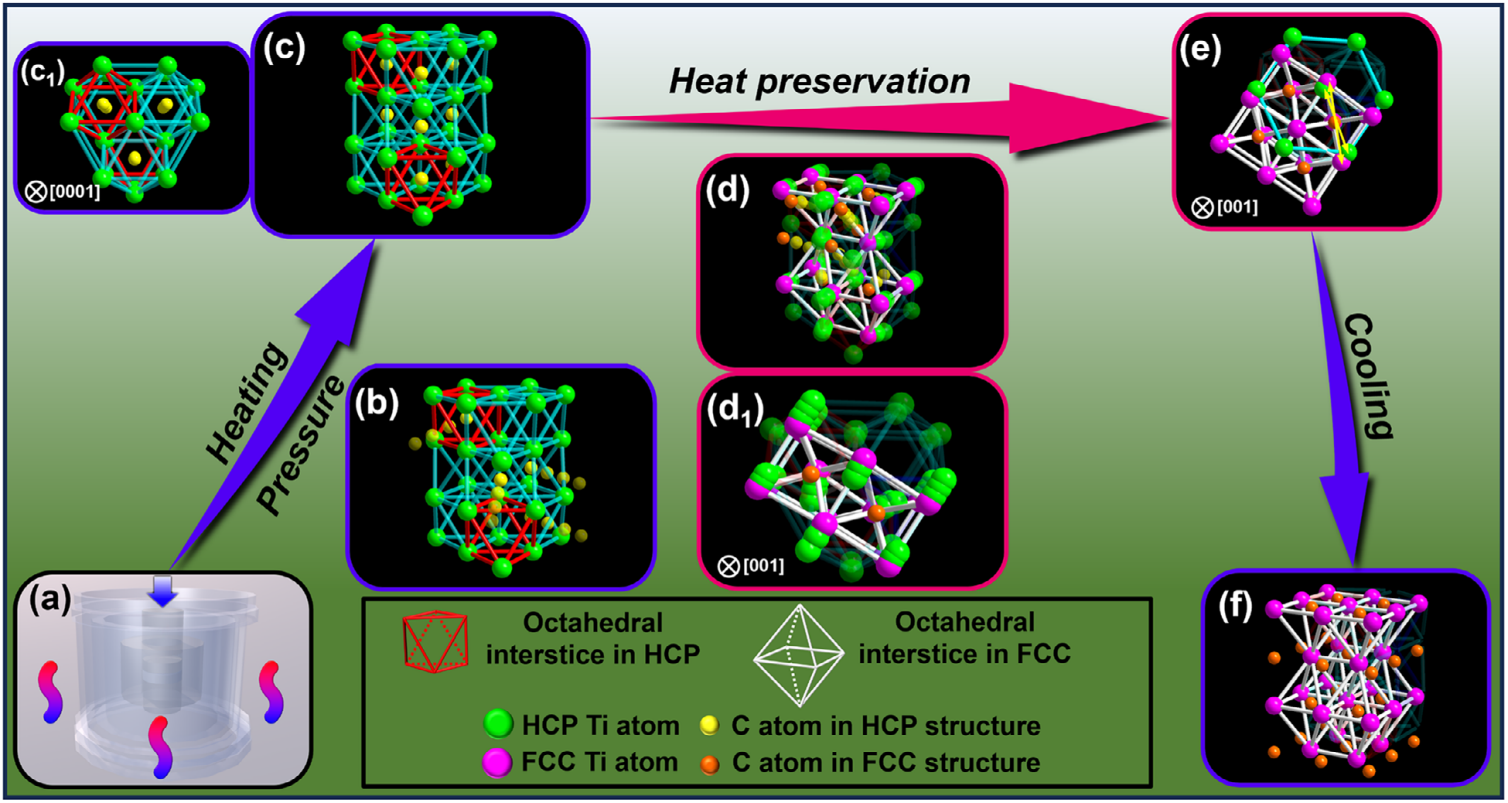
Schematic of TiC crystal phase formation mechanism under hot pressing.

Thus, two possible transformation mechanisms for TiC formation are proposed in this study. The first mechanism is predominantly driven by the intrinsic phase transformation of Ti, during which C atoms occupy octahedral interstitial sites in the lattice; the second mechanism is the structural transformation induced by C diffusion. Notably, both mechanisms are primarily driven by thermal pressure, and thus, it can be concluded that to exhibit a synergistic positive effect on the formation of the TiC crystal phase. Accordingly, a co‐induction mechanism of TiC crystal phase formation through C diffusion and phase transformation under thermal pressure is proposed, as shown in **Figure** **5**. Under high‐temperature and pressure conditions, the interface initially undergoes heating (Figure 5a). During this stage, both Fe and C atoms diffuse into the Ti matrix. However, owing to their higher diffusion rate, the C atoms preferentially occupy the octahedral interstices in the HCP‐Ti lattice (Figure 5b).^[^ ^34^ ^]^ The number of C atoms diffusing into the Ti lattice remains relatively low, and the external energy is insufficient to overcome the energy barrier and induce a structural transformation. Consequently, the C atoms are temporarily stabilized within the octahedral interstices in the HCP‐Ti (Figure 5c, the top view is shown in Figure 5c, 1). However, this state is unstable. During the holding stage (Figure 5d, the top view is shown in Figure 5d_1_), as the temperature and pressure reach the set values, the number of C atoms diffusing into the Ti lattice increases; thus, the HCP structure gradually becomes regionally unstable. At this juncture, both the Ti and C atoms undergo significant displacement. The Ti atoms migrate toward the higher‐stability FCC unit cell and occupy face‐centered positions, whereas the C atoms enter the octahedral interstices in the emerging FCC structure (Figure 5e, indicated by a yellow arrow). Upon completion of this process, the atomic arrangement reaches a stable state, resulting in the formation of a core FCC‐TiC structure. During the cooling stage, the crystal grew and eventually formed a thermodynamically stable TiC with an FCC structure (Figure 5f).

## 3 Conclusion

The crystal structure and formation mechanism of TiC, a typical reaction product that forms at the interface of TA2 pure Ti and 45# carbon steel under solid‐state diffusion conditions, were investigated in this study. Using the iDPC technique, this study experimentally confirmed, for the first time, that C atoms are stable in the octahedral interstices in FCC‐TiC under equilibrium conditions. This discovery overcomes the limitations of current theoretical model‐based speculations and provides significant experimental support for an in‐depth understanding of the TiC formation mechanism. Based on the findings of this study, it is speculated that FCC‐TiC formation is caused by the combined effect of C diffusion and Ti phase transformation during the hot‐pressing process. The proposed mechanism was used to elucidate the crystal phase evolution process from HCP‐Ti to FCC‐TiC.

The results of this study are significant for both theoretical and practical applications, as they provide a scientific basis for the design and regulation of high‐quality steel–Ti interfaces. Moreover, TiC typically exhibits non‐chemical stoichiometric characteristics, primarily owing to the incomplete occupation of the octahedral interstices by C atoms in the lattice. This phenomenon may be closely related to the diffusion of C atoms. By regulating the distribution of C vacancies in TiC, customized material performance for specific applications can be achieved. Notably, TiC with different stoichiometric ratios exhibits significant variations in hardness, electrical conductivity, and thermal conductivity,^[^ ^22^, ^35^ ^]^ with expected applications in neural electrode interface materials.^[^ ^36^ ^]^ Furthermore, the vacancies in the TiC crystal structure have broad application prospects in the field of energy storage, especially for hydrogen storage.^[^ ^34^, ^37^, ^38^ ^]^ Therefore, the results of this study are expected to introduce new research prospects in these fields, thereby enabling the fabrication of unique systems for advanced applications.

## 4 Experimental Section

### Experimental Design and Specimen Fabrication

**Figure 6:**
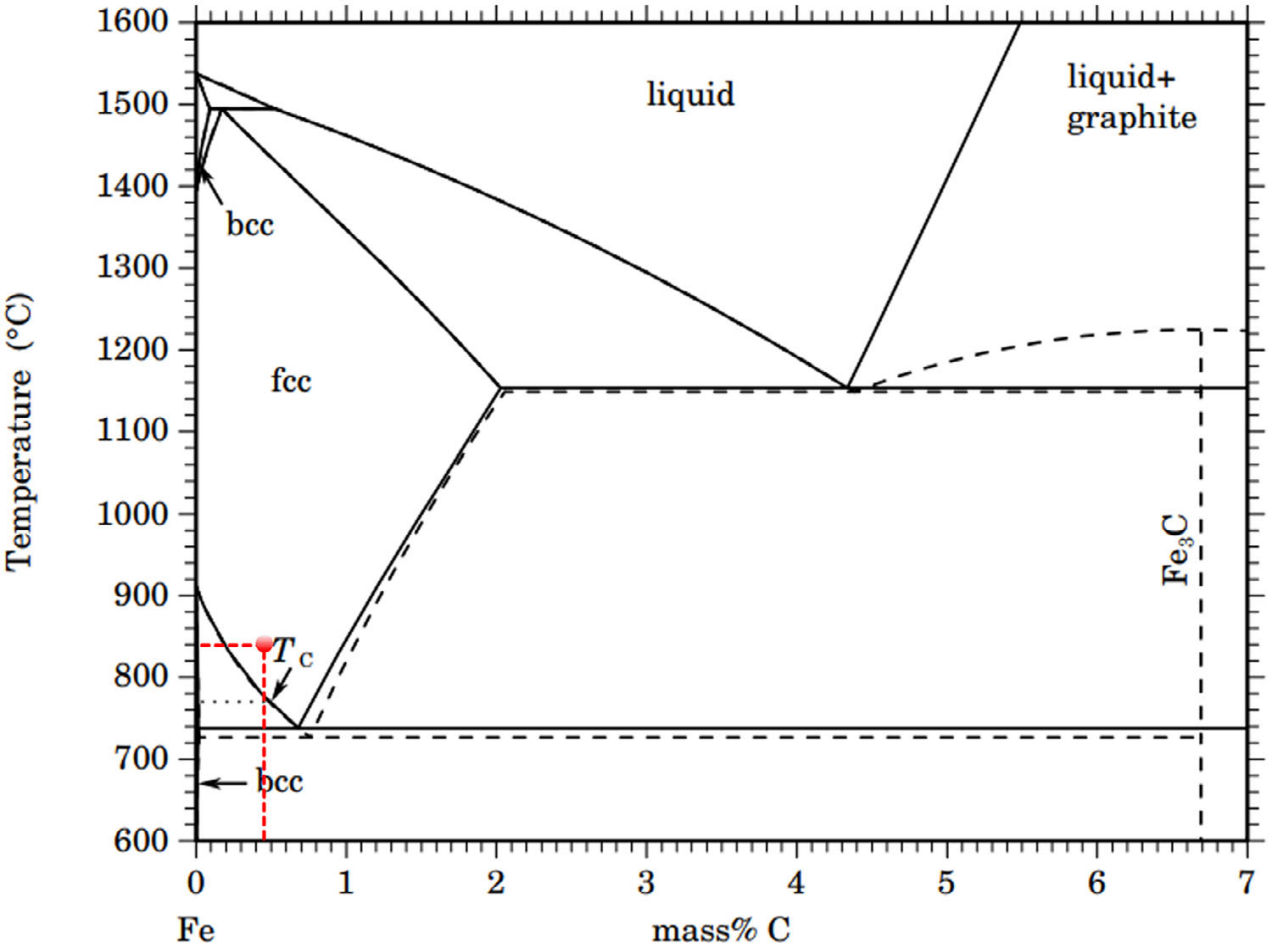
Fe‐Fe_3_C phase diagram.^[^ ^39^ ^]^

**Figure 7:**
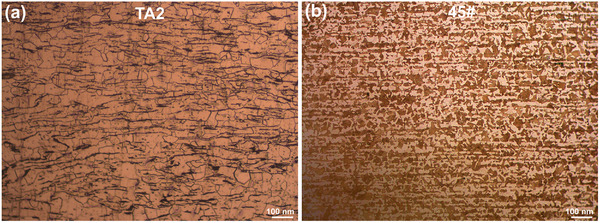
Metallographic structures of original base materials: a) TA2 pure Ti and b) 45# carbon steel.

Experiments were conducted under vacuum hot‐pressing conditions using specimens of industrial‐grade TA2 pure Ti and 45# carbon steel (Φ47 × H12 mm) as base materials. High temperatures and pressures were applied to facilitate mutual contact and diffusion between the Ti and carbon steel. This control method had two primary advantages. First, temperature regulates the form of C within carbon steel: at temperatures below the eutectoid temperature (≈720 °C), C predominantly exists in the compound state; however, it transitions to a solid solution state at temperatures exceeding the eutectoid temperature (**Figure** **6**). Further, a higher diffusion rate and reactivity were obtained for C in the solid solution state than in the compound state; therefore, the diffusion reaction between C and Ti was enhanced at high temperatures. Second, the kinetics of the interfacial reaction could be controlled by adjusting the holding time, thereby ensuring TiC nucleation and growth close to equilibrium. For the experiment conducted in this study, the aim was to control the temperature to enable C atom diffusion in the solid solution state and ensure complete nucleation and growth of the TiC formed at the interface. Consequently, an experimental temperature of 840 °C was set for 80 min under 35 MPa pressure, referencing the Fe–Fe_3_C phase diagram shown in Figure 6. The metallographic structures of the original Ti and carbon‐steel base materials along the axial direction are shown in **Figure** **7**a and b, respectively. Distinct horizontal distribution characteristics along the rolling direction were observed for both base materials. The chemical compositions of the base materials are summarized in **Table** **1**.

**Table 1:** Chemical compositions of base materials (wt.%).

Elements Base materials	Fe	Ti	C	O	S	Si	Mn
TA2	0.29	Bal.	/	0.16	0.01	/	/
45#	Bal.	/	0.46	/	0.02	0.18	0.56

**Figure 8:**
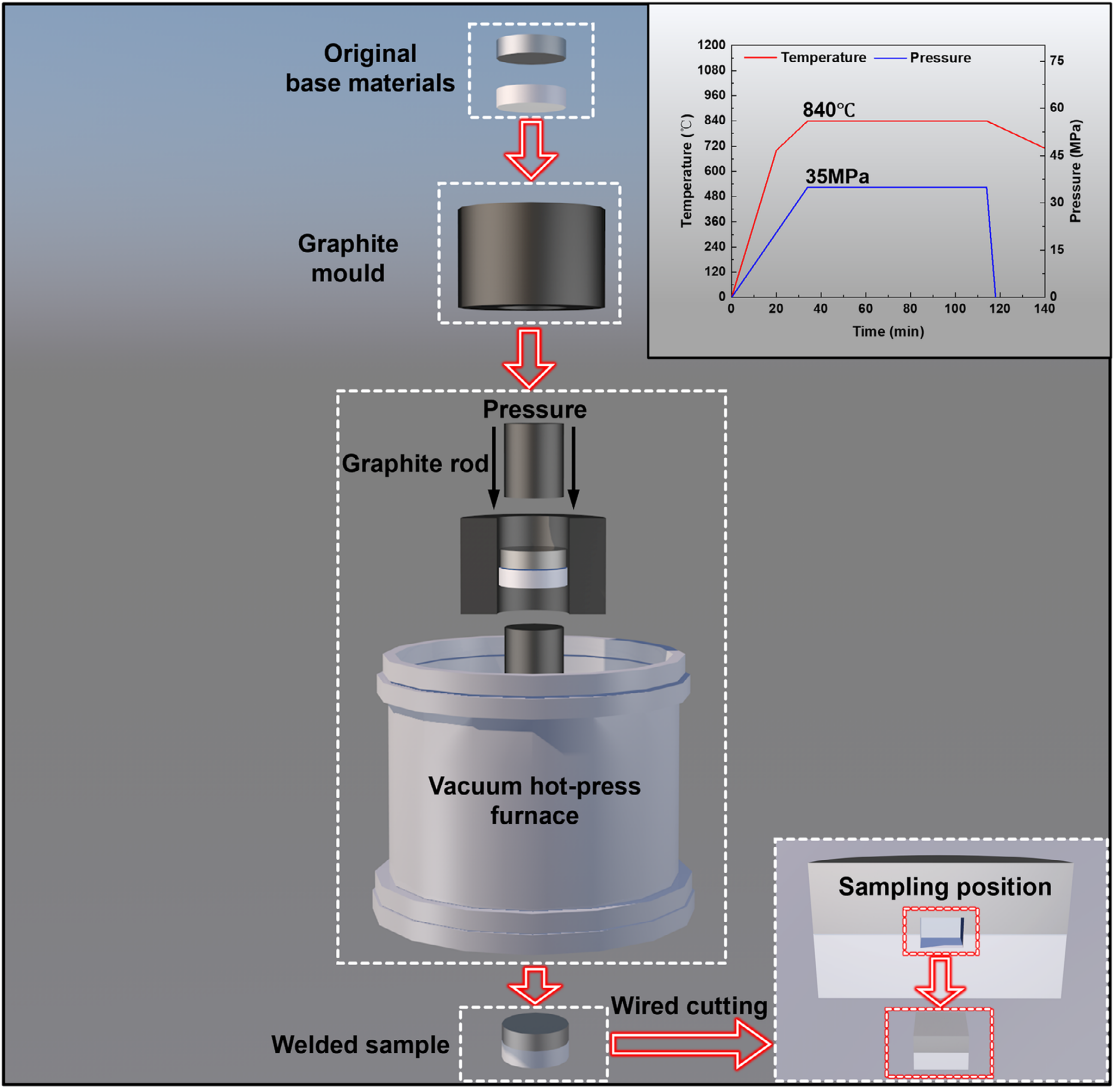
Sample preparation process.

Prior to diffusion bonding, the surfaces of the Ti and steel base materials were sanded and polished to a mirror finish. Then, the base metals were positioned within a graphite mold (following the assembly method shown in **Figure** **8**), which was placed in a vacuum hot‐press furnace. Following the holding process, the samples were cooled in the furnace and then removed. The process curves showing the changes in temperature and pressure over time are provided in the upper‐right corner of Figure 8. Finally, the diffusion‐bonded sample was wire‐cut to dimensions of 8 × 8 × 5 mm^3^ for focused ion beam (FIB) sampling (lower‐right corner of Figure 8).

### Characterization Methods and Simulation

**Figure 9:**
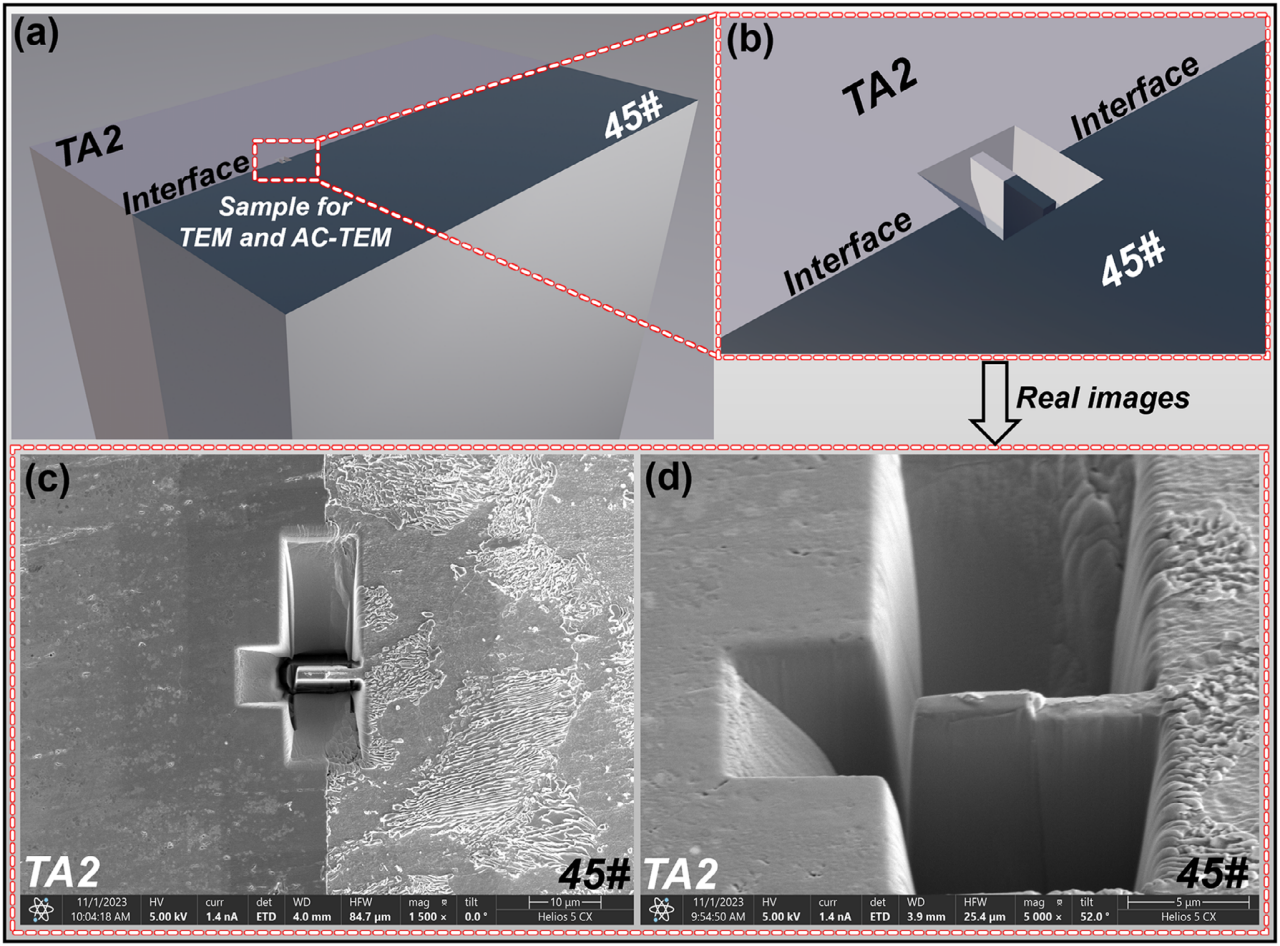
Sampling locations for characterization: a,b) schematics, and c,d) scanning electron microscope images.

The morphology, distribution, and chemical composition of the TiC formed at the interface were characterized using transmission electron microscopy. The crystal‐structure characteristics of the TiC were further examined using AC‐TEM. The C‐atom occupancy within the TiC lattice was investigated using iDPC technology. The samples for characterization were prepared by applying FIB at the interface. **Figure** **9**a,b show schematics of the interface and sampling positions, and Figure 9c,d show scanning electron microscope images of the sampling positions observed from different angles. The factors influencing the TiC crystal phase evolution were verified and analyzed using molecular dynamics simulation software (LAMMPS). The modified embedded atom method (MEAM) potential developed by Kim et al.^[^ ^40^ ^]^ was used to model the interactions between two Ti atoms. An initial volume of 20 × 20 × 20 Å^3^ was designated for HCP‐Ti, and periodic boundary conditions were implemented in the *x*‐, *y*‐, and *z*‐directions. The system energy was minimized using the conjugate gradient method; hence, a reasonable HCP structure was obtained. Subsequently, the system was fully relaxed within an NVT ensemble to ensure equilibrium at the target temperature prior to loading. Following relaxation, the entire system was maintained at a constant temperature using an NPT ensemble, under which pressure was applied to the (0001) surface. Finally, the simulation data were characterized and analyzed using the OVITO open‐source visualization tool. To describe the interactions between the Ti and C atoms, the cohesive energies of various crystal structures were computed via LAMMPS using the MEAM potential.^[^ ^41^ ^]^ Prior to the cohesive‐energy calculations, the various systems were fully relaxed and their energies were minimized. Finally, the calculated values would be directly output by the computer.

## Conflict of Interest

The authors declare no conflict of interest.

## Author Contributions

S.Z. wrote the original draft, –wrote, reviewed, and edited the final draft, performed investigation, formal analysis, and data curation. G.Y. was responsible for supervision, methodology, conceptualization, formal analysis, and funding acquisition. Y.Y. contributed to writing (review and editing), investigation, and data curation. L.D. was involved in writing (review and editing), methodology, investigation, and formal analysis. C.G. contributed to visualization, resources, validation, and software. B.J. was responsible for writing (review and editing), supervision, project administration, conceptualization, and funding acquisition.

## Data Availability

The data that support the findings of this study are available from the corresponding author upon reasonable request.
